# Natural Sunlight Shapes Crude Oil-Degrading Bacterial Communities in Northern Gulf of Mexico Surface Waters

**DOI:** 10.3389/fmicb.2015.01325

**Published:** 2015-12-01

**Authors:** Hernando P. Bacosa, Zhanfei Liu, Deana L. Erdner

**Affiliations:** Marine Science Institute, The University of Texas at AustinPort Aransas, TX, USA

**Keywords:** deepwater horizon, bacterial community, sunlight, oil pollution, Corexit, Gulf of Mexico, biodegradation, photooxidation

## Abstract

Following the Deepwater Horizon (DWH) spill in 2010, an enormous amount of oil was observed in the deep and surface waters of the northern Gulf of Mexico. Surface waters are characterized by intense sunlight and high temperature during summer. While the oil-degrading bacterial communities in the deep-sea plume have been widely investigated, the effect of natural sunlight on those in oil polluted surface waters remains unexplored to date. In this study, we incubated surface water from the DWH site with amendments of crude oil, Corexit dispersant, or both for 36 days under natural sunlight in the northern Gulf of Mexico. The bacterial community was analyzed over time for total abundance, density of alkane and polycyclic aromatic hydrocarbon degraders, and community composition via pyrosequencing. Our results showed that, for treatments with oil and/or Corexit, sunlight significantly reduced bacterial diversity and evenness and was a key driver of shifts in bacterial community structure. In samples containing oil or dispersant, sunlight greatly reduced abundance of the Cyanobacterium *Synechococcus* but increased the relative abundances of *Alteromonas, Marinobacter, Labrenzia, Sandarakinotalea, Bartonella*, and *Halomonas*. Dark samples with oil were represented by members of *Thalassobius, Winogradskyella, Alcanivorax, Formosa, Pseudomonas, Eubacterium, Erythrobacter, Natronocella*, and *Coxiella*. Both oil and Corexit inhibited the *Candidatus* Pelagibacter with or without sunlight exposure. For the first time, we demonstrated the effects of light in structuring microbial communities in water with oil and/or Corexit. Overall, our findings improve understanding of oil pollution in surface water, and provide unequivocal evidence that sunlight is a key factor in determining bacterial community composition and dynamics in oil polluted marine waters.

## Introduction

The Deepwater Horizon (DWH) oil spill was the largest offshore oil spill in history and released over 4.9 million barrels of crude oil to the Gulf of Mexico (Crone and Tolstoy, 2010). One of the strategies employed to mitigate the environmental impacts of the spill was the application of about 7 million liters of Corexit 9500 dispersant by planes and small vessels on the surface water, and injection to the wellhead at a water depth of 1500 m (TFISG, 2010). A massive amount of oil also ascended to the upper surface water forming numerous vast slicks and sheens in the northern Gulf of Mexico (nGoM) (Klemas, 2010).

Indigenous oil-degrading microorganisms play a critical role in the degradation of the spilled oil (Atlas and Hazen, 2011). Biodegradation was found to be an important process for the fate of oil in the deep water plume (Camilli et al., 2010; Hazen et al., 2010; Valentine et al., 2010; Redmond and Valentine, 2012; Dubinsky et al., 2013). While several studies have focused on the oil plume, little is known on the microbial degradation of hydrocarbons on the surface water under the relevant conditions in the nGoM. The sea surface is a more complex environment where dissolution, dispersion, emulsification, evaporation, biodegradation, and photochemical degradation all occur, often simultaneously. In particular, the nGoM sea surface is characterized by high water temperatures (28–30°C) and strong solar irradiance, which could have affected the fate of oil and the development of the microbial communities during the DWH spill (Liu et al., in press).

The effects of natural or artificial sunlight on bacterial activities in surface water vary remarkably and depend on the interplay of organic matter, aquatic microorganisms, and length of exposure (Carlucci et al., 1985; Medina-Sánchez et al., 2002; Santos et al., 2012; Ruiz-González et al., 2013). Ultraviolet (UV) radiation affects the structure of estuarine microbial communities (Santos et al., 2012), especially when a substantial amount of organic matter is oxidized (Hunting et al., 2013). In unpolluted waters, sunlight plays a relevant, yet difficult to predict, role in the community structure and function of heterotrophic bacteria, because other environmental factors such as nutrient availability and temperature modulate the interaction between bacteria and sunlight (Hunting et al., 2013; Ruiz-González et al., 2013). In oil polluted waters, crude oil adds to the carbon pool, and aromatic hydrocarbons absorb UV light that has a crucial role in the long-term weathering of spilled oil (Payne and Phillips, 1985; Evdokinmov and Losev, 2007). Aromatic hydrocarbons are more sensitive to photooxidation and generally transform into polar species (Garrett et al., 1998; Dutta and Harayama, 2000; Prince et al., 2003; Bobinger and Andersson, 2009; King et al., 2014; Bacosa et al., 2015). The presence of oil and Corexit dispersant in surface water may also affect the bacterial communities tremendously as oil is a complex mixture of aliphatic and aromatic hydrocarbons, which provides additional carbon and energy source or is toxic to the microbiota (Head et al., 2006; Hamdan and Fulmer, 2011; Kujawinski et al., 2011; Chakraborty et al., 2012).

When exposed to sunlight, oil collected from the surface of the Gulf of Mexico following the DWH spill produced substantial amounts of hydroxyl radical (Ray and Tarr, 2014a), singlet oxygen (Ray and Tarr, 2014b), and various oxygenated compounds (Ray et al., 2014). Metabolites from PAHs such as polar quinones are also formed when oil is irradiated (Arfsten et al., 1996; Holt et al., 2005). Although, these intermediate products are more soluble in water than the parent hydrocarbons, they are known to be reactive species that cause oxidative stresses, damage cells, thus are more toxic (Arfsten et al., 1996; Bertilsson and Widenfalk, 2002). The Corexit 9500 is a mixture of hydrocarbons, glycols, and dioctylsulfosuccinate (Chakraborty et al., 2012). Under simulated sunlight many of these components are photodegraded mainly through indirect photolysis via hydroxyl radical (Batchu et al., 2014; Glover et al., 2014; Kover et al., 2014).

Despite the frequency of marine oil spills, no study has examined how natural sunlight modulates bacterial community structure in oil-polluted waters. The research to date has primarily focused on polycyclic aromatic hydrocarbons (PAHs), which do not represent the complexity of spilled oil. UV irradiation of PAHs creates toxic metabolites and other reactive species that can inhibit bacterial growth and metabolism (Bertilsson and Widenfalk, 2002); for example phenanthrenequinone, a photooxidation product of phenanthrene, inhibited naphthalene degradation by *Burkholderia* (Holt et al., 2005). In a dark incubation, Edwards et al. (2011) demonstrated that bacteria in offshore oligotrophic surface waters near the DWH site rapidly degraded the oil, but the bacterial growth was limited by phosphate. Liu et al. (in press) showed that oil in surface water samples from nGoM underwent rapid weathering in 3 months, but they did not examine temporal changes in microbial community composition in those samples. Members of *Alphaproteobacteria* were the prevailing groups in oil mousses collected on sea surface during the DWH spill (Liu and Liu, 2013), but we do not know whether their presence relates to hydrocarbon exposure, high temperatures, strong irradiance, or a combination of these factors.

We hypothesized that sunlight was a contributing factor in shaping microbial community structures in oil-polluted waters during the DWH oil spill. To test this hypothesis, we examined the effect of natural solar radiation on microbial community dynamics in oil-polluted water in the presence and absence of Corexit dispersant. We used surface water collected near the DWH site in May 2013 and incubated it under the conditions of natural sunlight and temperature that are representative of the nGoM. Here we specifically addressed the question: How might sunlight have affected the bacterial community in oil-contaminated waters during the DWH spill?

## Materials and methods

### Seawater sampling and experiment set-up

This experiment was conducted using the same surface water, under similar condition (sunlight and temperature), and in parallel to our work on the biodegradation and photooxidation of oil (Bacosa et al., 2015). Briefly, in May 2013, seawater was collected from 0 to 2 m depth near the DWH site (28.74°N, 88.36°W) using Niskin bottles mounted on a conductivity-temperature-depth (CTD) array deployed from the R/V *Pelican*. The temperature, salinity, and dissolved oxygen were 25.1°C, 35 ppt and 6.6 mg L^−1^ (Liu and Liu, 2015). The water from several Niskin bottles was mixed in one sterile carboy and used for both this experiment and the degradation experiment of Bacosa et al. (2015). Amber bottles (500-mL) were used for dark treatments and 1000-mL quartz bottles for light treatments. Using a sterile graduated cylinder, 500 mL were transferred to 1000-mL quartz bottles and 250 mL was transferred to 500-mL amber glass bottles from the well-mixed water sample from the carboy. All bottles were capped well and sealed with parafilm to avoid contamination. Bottles for dark treatment were wrapped with aluminum foil. Bottle size and water volume have been shown to have negligible effects on microbial growth (Hammes et al., 2010). Nonetheless, all bottles started at the same headspace: seawater volume and surface area: volume, and sampling volumes were designed to maintain equal ratios in the dark and light bottles throughout the experiment.

The experimental treatments included four dark treatments and four light treatments (Table 1). Control treatments contained seawater only. Dispersant treatments were amended with Corexit 9500A at a final concentration of 10 ppm. Oil treatments were amended with Light Louisiana Sweet (LLS) crude oil at a final concentration of 200 ppm, which was the average total petroleum hydrocarbon (TPH) concentration detected in surface water after the DWH spill (Sammarco et al., 2013). Oil+dispersant treatments contained LLS and Corexit at a ratio of 20:1 (200:10 ppm). This ratio was equivalent to the nominal rate of dispersant application at the time of spill and within the range recommended by U.S EPA (EPA, 1995; Dispersant Aerial Application Systems: Airborne Support Incorporated, 2014). LLS was provided by BP as a surrogate for Macondo Oil MC252. Bottles for dark treatments were wrapped with aluminum foil. Two replicate bottles were prepared for each treatment. The hydrocarbons in the starting seawater sample were below detection limit. Corexit dispersant was not measured in the samples.

**Table 1:** **Description of experimental treatments**.

	**Treatment**	**Description**
1	Dark-seawater	Seawater only
2	Dark-dispersant	Seawater + Corexit 9500A
3	Dark-oil	Seawater + Light Louisiana Sweet crude oil (LLS)
4	Dark-oil+dispersant	Seawater + LLS + Corexit 9500A
5	Light-seawater	Seawater only
6	Light-dispersant	Seawater + Corexit 9500A
7	Light-oil	Seawater + Light Louisiana Sweet crude oil (LLS)
8	Light-oil+dispersant	Seawater + LLS + Corexit 9500A

*Light Louisiana Sweet crude oil and Corexit 9500A were added at final concentration of 200 and 10 ppm, respectively*.

### Incubation and sampling

The incubation bottles were transferred to wire holder and placed in a rectangular incubation tank with inlet and outlet for flowing seawater. Incubation was initiated aboard R/V Pelican (0–3 days) and continued at the Pier Laboratory of the University of Texas Marine Science Institute in Port Aransas, TX (4–36 days). All bottles were manually shaken lightly for about 5 s every day. Incubation was conducted under natural sunlight and with flowing surface seawater to maintain the ambient seawater temperature from May to July 2013. From 0 to 3 days flowing seawater from offshore nGOM was used, and from 4 to 36 days surface water from the Port Aransas ship channel was directly supplied to the incubation tank. The water was circulated around the sealed bottles to maintain ambient temperature. The average water temperature was 28°C. The average irradiance from 4 to 36 days was determined to be 250, 550, and 480 μmol photons/m^2^/s from the measurements taken at 7–8 a.m., 12–1 p.m., and 4–5 p.m., respectively (Figure S1).

Subsamples for microbial enumeration and community analysis were taken from duplicate bottles at 5, 10, 20, 27, and 36 days. The initial sample was obtained from the well-mixed surface water in the carboy before the start of the experiment (day 0). After lightly mixing the bottles, 100 and 50 ml of water were sampled using sterile pipettes from the light and dark treatments, respectively, and transferred to sterile flasks for subsequent processing. For DNA extraction, about 50 ml of water from each replicate was filtered through a 0.20 μm polycarbonate membrane filter using a vacuum pump. The filters with cells were stored in sterile petri dishes at −20°C.

### Microbial enumeration

Samples for enumeration of total bacterial cells were preserved in formaldehyde at a final concentration of 2% and stored at 4°C until analysis. Bacterial cells were stained with SYBR Green and counted using a flow cytometer (BD Accuri C6) as previously described (Liu et al., 2013). The most probable number (MPN) method was used to estimate the total aliphatic and aromatic hydrocarbon degrading bacteria using a modified protocol of Wrenn and Venosa (1996). Briefly, aliquots from the duplicate samples were pooled together and serially diluted in a saline buffer solution containing 0.1% sodium pyrophosphate (pH 7.5) and 2% NaC1. To each well of a 96-well microtiter plate, 20 μL of the diluted sample and 180 μL of Bushnell Haas Medium (Sigma-Aldrich, Inc) were added. Lastly, each well included either 10 μL of *n*-hexadecane, to test for aliphatic hydrocarbon degraders, or 9 μL of 5 mg mL^−1^ fluorene, 10 mg mL^−1^ phenanthrene, and 5 mg mL^−1^ pyrene, for PAH degraders. Alkane incubations were conducted for 2 weeks while PAH incubations for 3 weeks. Positive wells were scored after an overnight incubation with iodonitrotetrazolium violet (INT) at room temperature.

### DNA extraction and pyrosequencing

The membrane filters for bacterial community analysis were cut into small (1–2 mm) pieces using sterile scissors and transferred to 2 ml tubes. DNA was extracted from the filters using Powersoil DNA Isolation Kit (MO BIO Laboratories, Inc.) following the manufacturer's protocol, and quantified by UV spectrophotometry (GE NanoVue). DNA extracts from duplicate samples were pooled together in one tube.

The pooled DNA samples were used for bacterial community analysis via bacterial tag-encoded FLX amplicon pyrosequencing (bTEFAP). A~500 bp region of the 16S rRNA gene was amplified using Eubacterial primers 28F (5′TTTGATCNTGGCTCAG-3′) and 519 R(519R 5′-GTNTTACNGCGGCKGCTG-3′) (Dowd et al., 2008; Smith et al., 2010). Pyrosequencing was performed at the Research and Testing Laboratory (Lubbock, TX) using Roche 454 FLX instrument with Titanium Reagents according to the RTL protocols (www.researchandtesting.com) for bacterial diversity (Smith et al., 2010).

### Analysis of the sequence data

The raw data files were converted into FASTA files and analyzed by the Research and Testing bioinformatics pipeline consisting of four major stages—quality trimming, clustering, chimera checking, and denoising. Quality scores were used to clean up the potentially low-quality ends of each read by trimming. Reads were then classified into clusters using the USEARCH algorithm (Edgar, 2010), followed by chimera checking using UCHIME chimera detection software executed in *de novo* mode (Edgar et al., 2011). The final stage of the pipeline was denoising, which created quality sequences for use in taxonomic analysis pipeline. The denoised and chimera checked reads were then condensed into a single FASTA formatted file that contained reads from longest to shortest. These sequences were then clustered into operational taxonomic units (OTUs). The seed sequence for each cluster was queried against a database of high quality sequences derived from NCBI using.NET algorithm that utilized BLASTN+ (KrakenBLAST www.krakenblast.com). The database was developed by Research and Testing Laboratory (RTL) in Lubbock, Texas. The sequences were classified at the appropriate taxonomic levels based on the following criteria; greater than 97% for species level, 95–97% for genus level; 90–95% at the family level, 85–90% at order level, 80–85% at class and 77–80% at phylum level. The percent abundance of each organism was then calculated for each sample based upon the proportional number of reads.

### Statistical analyses

Non-metric multidimensional scaling (NMDS) was used to examine the overall patterns of bacterial community structure using Hellinger-transformed relative abundances of bacterial genera. NMDS was performed using Bray-Curtis dissimilarity distances in PAST software package, V2.17 (Hammer et al., 2001). Treatments were then compared using one-way analysis of similarity (ANOSIM) to verify the significance of the clustering. Using Hellinger-transformed relative abundances, Principal component analysis (PCA) was also applied to the substrate-amended treatments to determine the bacterial genera that are associated with dark and light conditions. One-Way analysis of variance (ANOVA)was used to test for differences in the Shannon-Wiener Index (H) and Evenness (E) among treatments (PASW Statistic 18.0 (SPSS Inc., Chicago, IL, USA).

## Results

### Bacterial growth

**Figure 1:**
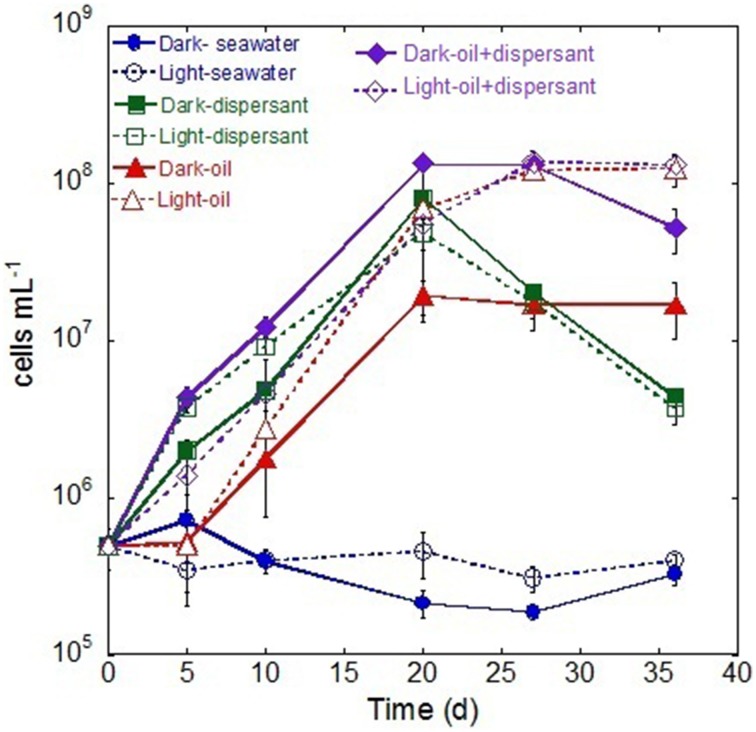
**Changes in bacterial density during the incubation in the dark and light conditions**. The values and error bars represents mean and standard deviation of two replicates, respectively. The bacterial cells were enumerated via flow cytometry.

During the first 5 days, only the treatments containing Corexit showed an increase in bacterial density (Figure 1), while those containing crude oil alone showed no growth. Between 5 and 20 days, cell densities in all samples with added substrates increased linearly, and cell numbers peaked at 20 days for most treatments. After 20 days, treatment with dispersant alone decreased abruptly (about 15-fold), while those with oil either increased slightly or plateaued. Throughout the whole incubation period, there were no marked changes of cell density in either light or dark control.

The MPN data showed similar dynamics to the flow cytometry measurements. The MPN counts revealed that the abundance of alkane degraders increased during the incubation between 5 and 20 days, after which they declined (Figure S2A). Dark treatment with both oil and Corexit yielded the highest density of alkane degraders. In general, PAH degraders exhibited a lag during the first 10 days and peaked between 20 and 27 days. Dark treatments with added dispersant and oil again had highest densities (Figure S2B).

### Bacterial community dynamics and diversity

**Figure 2:**
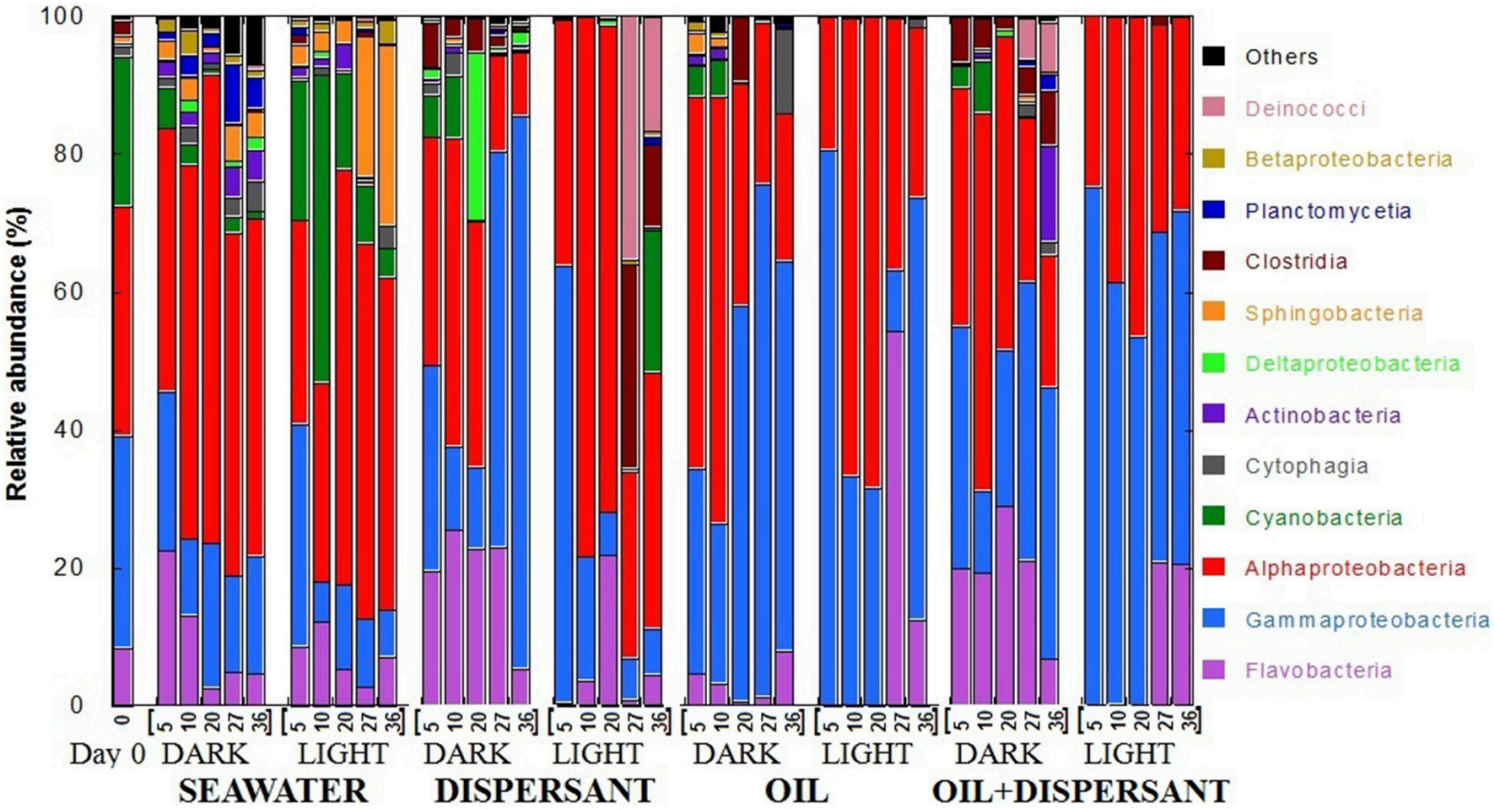
**Relative abundances of bacteria at class level in seawater alone, seawater with dispersant Corexit, crude oil, and both oil and dispersant incubated under the dark and light conditions for 36 days**. The numbers on the horizontal axis indicate the days of incubation.

Pyrosequencing of 16S rRNA gene amplicons from the 41 samples resulted in a total of 289, 066 quality-filtered sequences for an average of 7050 sequences per sample. At the class level, *Flavobacteria, Gammaproteobacteria, Alphaproteobacteria*, and *Cyanobacteria* dominated at almost all time points (Figure 2). The original surface seawater at the beginning of the experiment comprised roughly equal proportions of *Alpha*- (33%) and *Gammaproteobacteria* (31%), along with *Cyanobacteria* (22%) and *Flavobacteria* (8%). In controls with seawater alone, *Cyanobacteria* were greatly reduced in the dark, and *Sphingobacteria* increased by about 20-fold at the later stage of incubation under the light. The sequences were submitted to the Gulf of Mexico Research Initiative Information and Data Cooperative (GRIIDC) and are available in the URL http://data.gulfresearchinitiative.org/data/R1.x140.126:0005 and doi: 10.7266/N7H70CRW. The data were also submitted to NCBI Sequence Read Archive (SRA) under the Accession SAMN04054215. The relative abundances of bacteria genera is also available under doi: 10.7266/N7CF9N11.

The composition of bacterial communities varied over time between the dark and light treatments containing dispersant. During the first 20 days when sustained microbial growth was observed, dark-dispersant treatments contained mainly *Flavobacteria* (20–25%), *Gamma*- (12–31%) and *Alphaproteobacteria* (35%), whereas *Alphaproteobacteria* (78 and 70% at 10 and 20 days, respectively) dominated the light-dispersant samples. When bacterial density decreased at 27 and 36 days, *Gammaproteobacteria* dominated in the dark-dispersant treatment (60–80%), while *Clostridia* (12–29%) and *Deinococci* (17–25%) increased substantially in the light-dispersant treatment.

The oil and oil-dispersant treatments shared similar patterns. In general, the light samples were less diverse, almost exclusively *Alpha*- and *Gammaproteobacteria* during the growth period (until 20 days), then there was a burst of *Flavobacteria* after the peak density. Dark samples maintained some diversity of bacterial groups with higher proportion of *Flavobacteria* in dark-oil+disp compared to dark-oil. Notably, *Cyanobacteria* comprised 5–10% of community in the amended dark treatments until 10 days, but were barely detectable from 5 days in light treatments.

**Figure 3:**
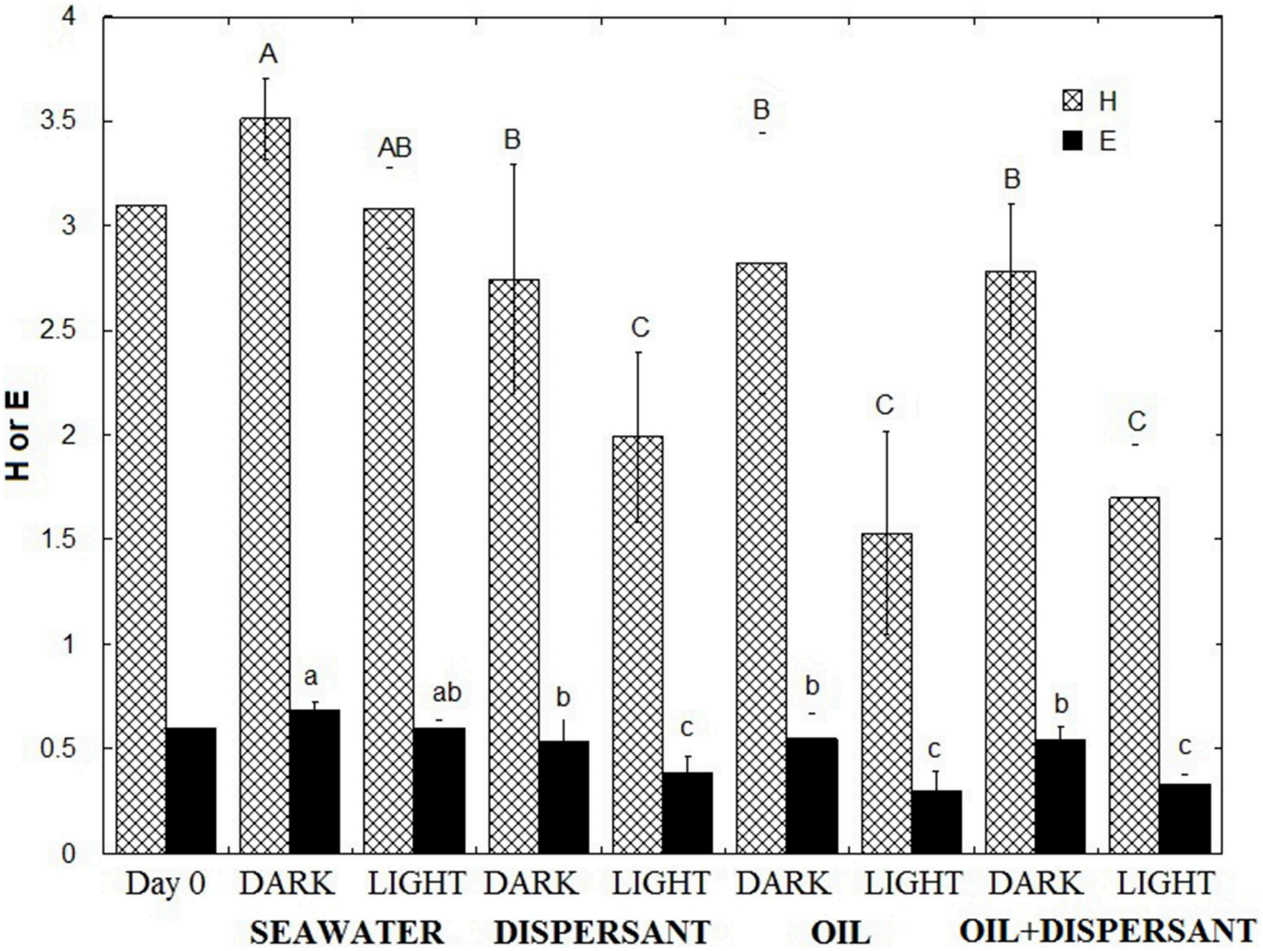
**Shannon Wiener Index (H) and Evenness (E) of microbial communities**. The indices represent the average of the time series (5, 10, 20, 27, 36 days). Error bar represents the standard deviation. Different letters are significantly different (*p* < 0.05) according to Tukey's HSD mean separation test.

To compare how the bacterial diversity varied among treatments, we calculated the mean Shannon-Wiener index of diversity (H) and evenness (E) of the five sampling points for each treatment using the relative abundances at the genus level (Figure 3). Comparison of the mean H of the eight treatments revealed that the highest bacterial diversity was found in the light- and dark-seawater controls, followed by the amended dark treatments. The light treatments showed the lowest diversity. One-way ANOVA revealed that values for light treatments were significantly lower than the dark treatments (*p* < 0.01). Tukey's-HSD test of the means further revealed that diversities in dark and light controls do not vary significantly. However, diversities of light treatments with added oil and/or dispersant were statistically lower than the corresponding dark treatments. The evenness of the community followed the same pattern as the diversity index; light treatments were significantly less even than dark treatments in amended bottles. Overall, natural sunlight significantly reduced microbial diversity and evenness in the presence of oil and/or Corexit dispersant.

### Effect of sunlight, oil, and dispersant

**Figure 4:**
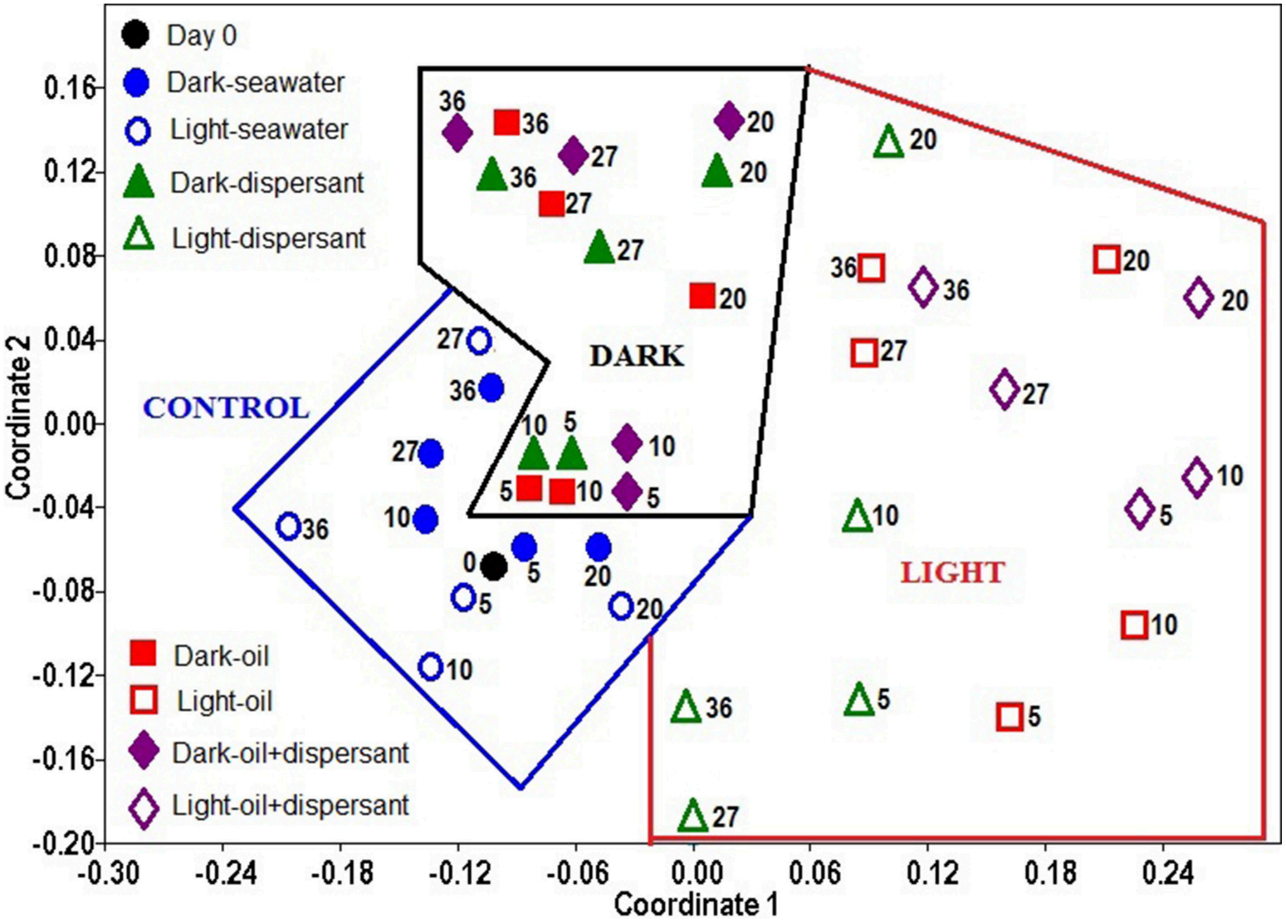
**Non- metric multidimensional scaling (NMDS) of bacterial community structures in light and dark conditions**. The ordination is based on Bray-Curtis coefficient matrix of relative abundances. The numbers indicate days of incubation.

The overall similarity of the microbial communities was examined with non-metric multidimensional scaling (NMDS), and the significance among treatments was further tested with ANOSIM using the bacterial genus data. The NMDS plot shows that the bacterial community structure in the control (seawater only) exhibited the closest similarity with the initial community (Figure 4). The 5 and 10 days amended dark treatment clustered closely to the control and initial community. The dark treatments at 20 days and later clustered together, separated from the light treatments and the seawater/control communities. All light treatments, from 5 days through 36 days, were apart from the dark and control samples, and they were more scattered, showing a greater variability throughout the incubation period under the natural sunlight.

ANOSIM confirmed that sunlight significantly affected bacterial community structure in seawater alone (*p* = 0.008) and in the presence of oil, dispersant, or oil+dispersant (*p* = 0.0001) (Table 2). ANOSIM also revealed that seawater control significantly differed (*p* < 0.001) from any of the amended treatments (dispersant, oil, oil+dispersant), but the three amended treatments do not vary from each other (*p* > 0.05). Testing the dark treatments and the light treatments separately revealed that dispersant, oil, and oil+dispersant incubated under the dark did not vary significantly. However, light-dispersant differed significantly from light with oil+dispersant (*p* = 0.016) but not from light-oil (*p* = 0.146). Also, the bacterial communities during the 5–20 days growth stage (peak growth) significantly differed from that of 27–36 days (*p* = 0.003), when the bacterial density decreased or plateau.

**Table 2:** **Pairwise comparison of bacterial communities in dark and light incubations based on ANOSIM with the Bray-Curtis distance (*P*-values)**.

	**R**	***p*-value**
Dark vs. light (seawater)	0.532	0.008^*^
Dark vs. light (substrates)	0.639	0.0001^*^
5–20 days vs. 27–36 days (substrates)	0.236	0.003^*^
Seawater vs. dispersant	0.383	0.0002^*^
Seawater vs. oil	0.394	0.0002^*^
Seawater vs. oil+dispersant	0.529	0.0001^*^
Oil vs. dispersant	0.142	0.076
Oil vs. oil+dispersant	−0.032	0.576
Dispersant vs. oil+dispersant	0.081	0.141
Light-dispersant vs. light-oil	0.192	0.146
Light-oil vs. light-oil+dispersant	−0.024	0.507
Light-dispersant vs. light-oil+dispersant	0.516	0.016^*^
Dark-dispersant vs. dark-oil	0.228	0.117
Dark-oil vs. dark-oil+dispersant	0.168	0.138
Dark-dispersant vs. dark-oil+dispersant	−0.080	0.636

Significant differences are marked by asterisk (*).

**Figure 5:**
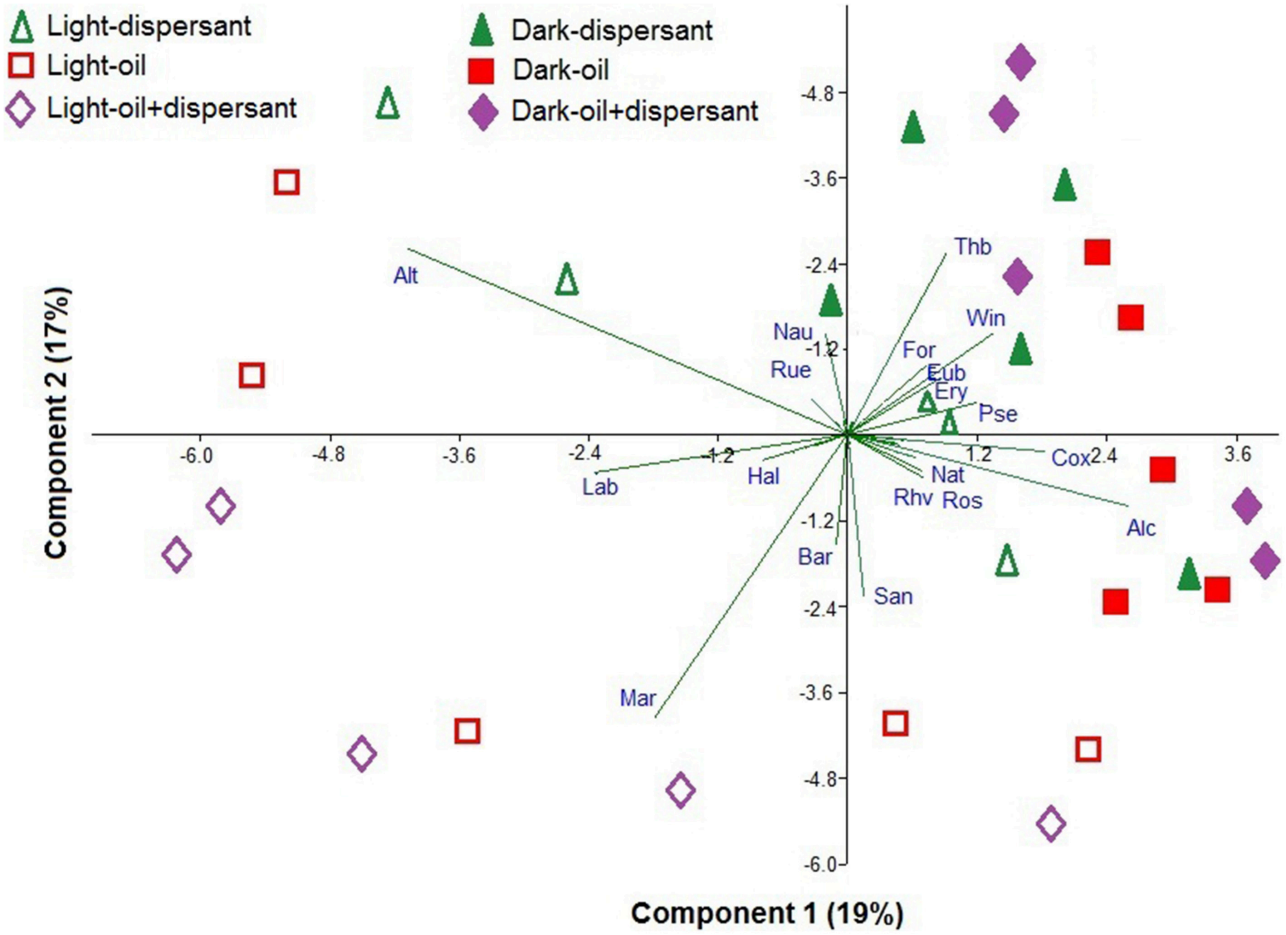
**Principal component analysis of the Hellinger-transformed relative abundances of genera in dispersant, oil, and oil+dispersant treatments**. The arrows represent the genus component loadings. Alt, *Alteromonas*; Lab, *Labrenzia*; Hal, *Halomonas*; Mar, *Marinobacter*; Bar, *Bartonella*; San, *Sandarakinotalea*; Rhv, *Rhodovulum*; Ros, *Roseobacter*; Nat, *Natronocella*; Alc, *Alcanivorax*; Cox, *Coxiella*; Pse, *Pseudomonas*; Ery, *Erythrobacter*; Eub, *Eubacterium*; Win, *Winogradskyella*; For, *Formosa*; Thb, *Thalassobius*; Nau, *Nautella*; Rue, *Ruegeria*.

Principal component analysis (PCA) was used to illustrate the bacteria that are associated with dark and light incubations (Figure 5). Diverse genera were associated with dark conditions; for example *Thalassobius, Winogradskyella, Alcanivorax, Formosa, Pseudomonas, Eubacterium, Erythrobacter, Natronocella*, and *Coxiella*, among others. On the other hand, a few genera were strongly associated with light conditions, including *Alteromonas, Marinobacter, Labrenzia, Sandarakinotalea, Bartonella*, and *Halomonas*.

**Figure 6:**
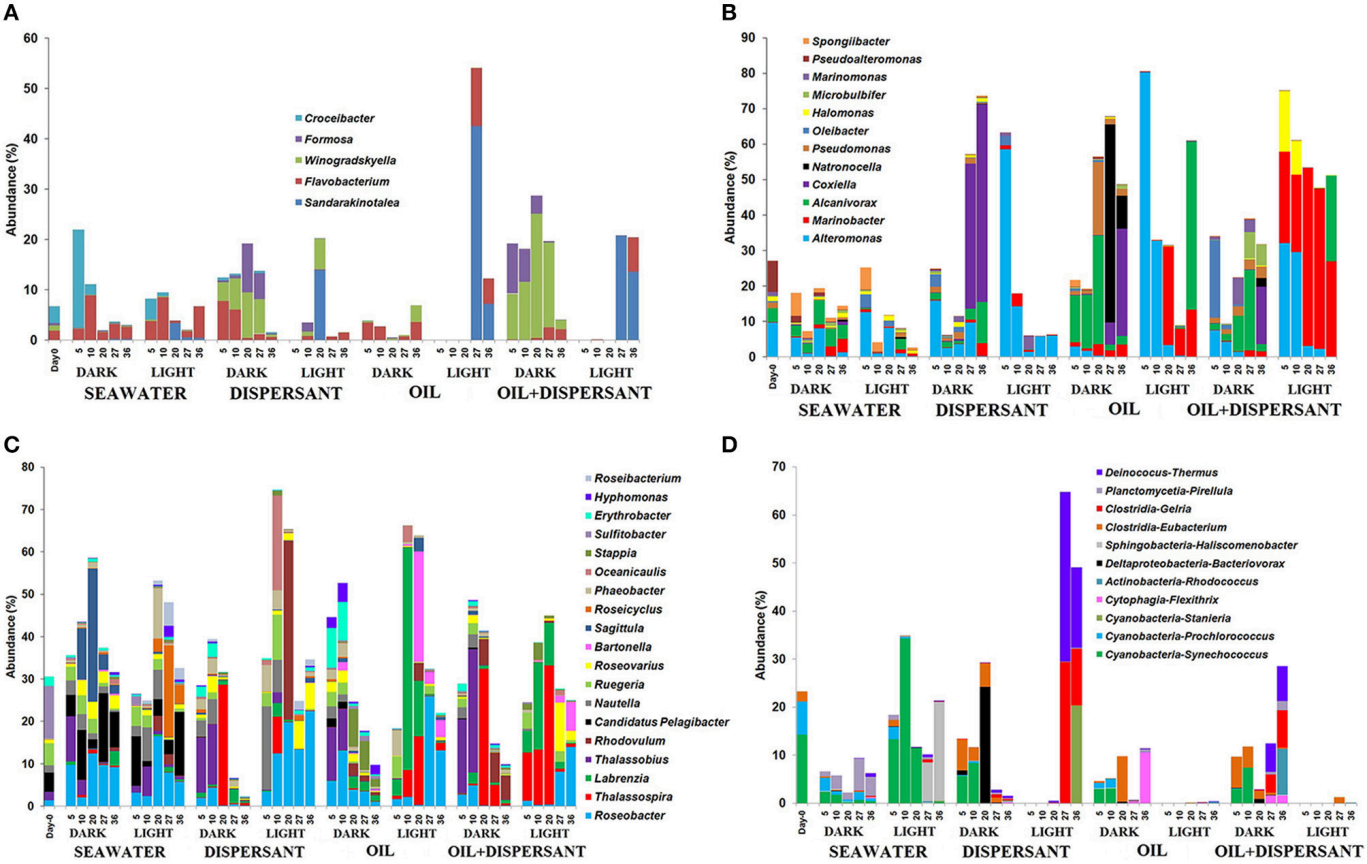
**Abundances of bacteria at genus level in seawater alone, seawater with dispersant Corexit, crude oil, and both oil and dispersant incubated under the dark and light conditions for 36 days**. Only abundances greater than 5% in at least one of the samples are shown. **(A)** *Flavobacteria*, **(B)** *Gammaproteobacteria*, **(C)** *Alphaproteobacteria*, **(D)** Other bacteria. The numbers indicate days of incubation.

Among the *Flavobacteria, Sandarakinotalea* became dominant in light treatments with oil after 27 days and in light-dispersant at 20 days (8–42%), while dark incubation favored *Winogradskyella* (5–25%) and *Formosa* (4–10%) in treatments with dispersant throughout the incubation period (Figure 6A). Of the members of *Gammaproteobacteria, Alteromonas* was represented in both light and dark treatments (Figure 6B). However, it dominated at the first 10 days in light treatments with amended substrates, constituting up to 60–80% of the total bacterial community. The abundance of *Marinobacter* (7–50%) was also greatly enhanced in light treatments with oil and oil+dispersant, and *Halomonas* (9–20%) in oil+dispersant. *Coxiella* predominated in later stage (27 and/or 36 days) of the dark-substrate treatments (10–55%). *Alcanivorax*, a known alkane degrader, was abundant in dark treatments with oil between 5 and 20 days (up to 30%), whereas in oil+dispersant samples, it increased between 20 and 36 days, In the light, *Alcanivorax* only became abundant at 36 days. *Natronocella* and *Pseudomonas* were also associated with dark incubation with oil.

*Alphaproteobacteria* comprised more than 30% of the initial community and most of the samples during incubation. Some differences in the community in seawater control were clearly observed between light and dark treatments (Figure 6C). For example, *Sagitulla* appeared in dark-seawater, while *Roseicyclus* appeared in light-seawater. However, dark and light treatments exhibited broad patterns with oil and Corexit addition. From barely detected at 0 days, *Roseobacter* and *Thalassospira* increased in both dark and light treatments at different times. On the other hand, *Thalassobius* (10–30%) exclusively occurred early (5–10 days) in all dark treatments. *Labrenzia* increased in light treatments with oil during the first 20 days (5–50%), while *Bartonella* appeared after 20 days (4–25%). *Rhodovulum* (42%) dominated at 20 days in light-dispersant, when bacterial density was higher, and appeared in dark-oil+dispersant (about 5%) during the later incubation period. Also, *Erythrobacter* (10%) increased in abundance during the first 10 days of incubation under the dark with oil alone. Furthermore, *Candidatus* Pelagibacter, an abundant member of SAR 11 clade was inhibited by all substrates in both dark and light conditions.

As expected, *Synechococcus* was reduced in dark-seawater and abundant in light-seawater (Figure 6D). Surprisingly, *Synechoccous* comprised 3–9% of the community until 10 days in dark treatments with added substrates, while it was not detected in light treatments. *Eubacterium* increased by 2- to 6-fold in dark treatments but was barely detected in light treatments. *Gelria* (4–30%) and *Thermus* (6–35%) predominated at the later stage in light with dispersant only, but not in light with both oil and dispersant.

## Discussion

This study aimed to evaluate the effects of natural sunlight on bacterial populations in surface seawater under the influence of crude oil, Corexit or both. To relate changes in bacterial community to the degradation of dispersed and non-dispersed oil, this was conducted in parallel to our previous work on the photooxidation and biodegradation of crude oil (Bacosa et al., 2015). There is good agreement in the microbial growth and oil degradation results. For example, the rapid increase in total cell density, alkane degraders and aromatic degraders from 10 to 20 days corresponded to the rapid disappearance of *n*-alkanes and PAHs (Figures S3A,C). Thus, the dominant members of the microbial communities (*Alteromonas, Alcanivorax, Marinobacter, Thalassospira, Thalassobius, Labrenzia*, and *Bartonella*) during these time points are likely key players in the degradation of hydrocarbon compounds in crude oil. Bacterial density increased linearly with dispersant alone both in dark and light. This suggests that bacteria utilized Corexit components for growth. Although, we have not analyzed Corexit or any of its components, recent studies showed that dioctyl sodium sulfosuccinate (DOSS), a major component of Corexit was rapidly degraded by bacteria from nGoM surface water within 8 days (Campo et al., 2013).

Aiming to enrich or isolate bacteria that utilize hydrocarbons as sole carbon and energy sources, culture-based studies do not usually consider the effect of sunlight and are typically performed in the dark (Head et al., 2006; Haritash and Kaushik, 2009; Bacosa et al., 2010; Das and Chandran, 2011; Edwards et al., 2011; Hamdan and Fulmer, 2011; Gutierrez et al., 2013b). We recently reported that sunlight was a predominating factor in the degradation of PAHs, while bacteria were key to degrading alkanes (Bacosa et al., 2015). Rapid degradation of alkanes in both light and dark treatments occurred between 10 and 20 days. With or without bacteria, PAHs were linearly degraded until 20 days when exposed to sunlight, while PAH degradation by bacteria in dark treatments occurred between 10 and 27 days. Although the biodegradation of aliphatic hydrocarbons in crude oil are very similar in the dark and light, and photooxidation is the major mechanism of PAH degradation (Bacosa et al., 2015), the current results suggest that different microbial communities developed in dark and light conditions. Moreover, the associated bacterial communities in Corexit treatment were different between light and dark treatments. The rapid degradation of alkanes between 10 and 20 days for both dark and light treatments suggests that even though the communities are different they can have very similar degradation capacity.

All light treatments differ significantly from dark treatments as well as from controls, which suggests that sunlight affects bacterial communities in marine surface waters that are contaminated by Corexit dispersant and/or crude oil (Figure 4, Table 2). Under natural sunlight, the community shifted further from dispersant to oil+dispersant, and that dispersant alone had lesser impact on bacterial community compared to that of oil and oil+dispersant (Figure 4). The communities during the growth stage (5–20 days) were also significantly distinct from that of 27–36 days. This was also reflected in the community structures at the genus level, suggesting that different bacteria were involved in the degradation of oil and Corexit, and other groups of bacteria may have utilized the metabolic products, as characterizable hydrocarbons were generally undetectable after 27 days (Bacosa et al., 2015). Although the light and dark bottles were different sizes, we observed no evidence of “bottle effects” in our incubations. Every bottle was filled halfway, thus the seawater: headspace was equivalent between the light and dark bottles. We maintained this ratio throughout our sampling, withdrawing more sample volume from the larger bottles. Previous studies have shown that volume does not affect microbial community structure during short-term (< 5 day) incubations (Hammes et al., 2010). We did not observe appreciable differences in microbial community structure between light and dark controls over the entire 36 day incubation. In contrast, significant changes in bacterial community structures occurred during the first 5 days for the oil/dispersant-treated samples, which suggests that bottle effects were not a significant driver of community structure. Further, incubation experiments in our lab show that volume does not affect peptide decomposition patterns (Liu et al., 2013), which agrees with previous observations that interactions of water with glass surfaces have minimal effects on bacterial function (Gardner et al., 1986). Overall, we do not think bottle effect contributed significantly to our results.

Redundancy analysis (RDA) on bacterial community data showed that light is the major driver of this change followed by crude oil, and then by dispersant (Figure S4). Partial RDA further revealed that sunlight explains almost 50% of the variation while oil and dispersant contributes about 30 and 20%, respectively (Table S1). Thus, sunlight is the dominant factor in shaping the microbial community in oil polluted surface water in the northern Gulf of Mexico, particularly during summer months, when a massive amount of oil was released in this area during the DWH oil spill (May-July).

Exposure to natural solar radiation resulted in a slight change in the microbial communities in seawater control, but tremendous shifts and decreases in bacterial diversity were observed in treatments amended with crude oil, Corexit, or both. This could result from enhanced toxicity of both oil and Corexit in light treatments through production of hydroxyl radicals or toxic metabolites that cause oxidative stress to the cells (Arfsten et al., 1996; Bertilsson and Widenfalk, 2002; Batchu et al., 2014; Glover et al., 2014; Kover et al., 2014; Ray et al., 2014; Ray and Tarr, 2014a). An alternative explanation for the reduced diversity in oil and Corexit-amended light treatments may be that the dominant bacteria in these treatments out-competed other bacteria, as sunlight is known to alter mutualistic and competitive interactions among aquatic microorganisms (Sommaruga, 2003). The accumulation of reactive species reduces the ability of the bacteria to compete, especially those with poor DNA repair capabilities (Häder et al., 2007). The microbial community in seawater changes with oil alone comprising several co-existing species that compete for a variety of chemically distinct hydrocarbons, each of which requires specific mechanism for activation and degradation (Yakimov et al., 2005; McGenity et al., 2012). As hydrocarbonoclastic bacteria rarely function in isolation but as a community in nature, this complex interaction is also not just between hydrocarbon-degraders but also between hydrocarbon- and non-hydrocarbon degraders (McGenity et al., 2012). For example, when PAH compounds are rapidly degraded in light treatments, bacteria that utilize metabolic products will overtake aromatic degraders. Some bacteria also release bioactive compounds that inhibit competitors (McKew et al., 2007). The nature of this competition however is rather complex and warrants more detailed study.

While sunlight negatively affected different phylotypes, it also enhanced certain bacteria. Recent studies revealed that in a bacterial community there may be UVR-tolerant phylotypes that have repair capabilities and react distinctly to light-driven processes (Ruiz-González et al., 2013). The light tolerance of some bacteria may be ascribed to photoheterotrophy (Karl, 2002; Zubkov, 2009), photoreactivesiderophores (Barbeau et al., 2001), and photosensory proteins and photoreceptors for DNA repair, stress response, and formation of biofilms (Van der Horst et al., 2007; Singh et al., 2009; Elías-Arnanz et al., 2011). Hydrocarbon-degrading bacteria are also associated with marine eukaryotic phytoplankton, which may provide the bacterial symbionts with an advantage during initial exposures to oil under light conditions (Gutierrez et al., 2013b, 2014). Proteorhodopsins, which are light-dependent proton pumps and widely distributed among surface water bacteria, provides energy to the cell and increases adaptation to environmental variability (DeLong and Béjà, 2010; Palovaara et al., 2014). Proteorhodopsin-coding genes are also present in ubiquitous alphaproteobacterial clades SAR11 that includes *Candidatus* Pelagibacter (Giovannoni et al., 2005). In this study, *Candidatus* Pelagibacter were inhibited in both dark and light suggesting that this bacteria likely played no role in the degradation of oil and dispersant.

Crude oil and dispersants had pronounced effect on *Candidatus* Pelagibacter and Cyanobacteria. Crude oil and/or dispersant inhibited *Candidatus* Pelagibacter regardless of lighting condition, supporting previous findings that this group of bacteria is particularly susceptible to oil pollution (Chronopoulou et al., 2015). However, this is the first report on the effect of Corexit dispersant on these ecologically and biogeochemically important bacteria. The Cyanobacteria *Synechococcus* and *Prochloroccus* were particularly inhibited in oil and Corexit-amended light treatments. From the analysis of near shore water microbial communities in the nGoM, Widger et al. (2011) reported that *Synechococcus* dramatically decreased when oil reached the sampling areas. Also, *Synechococcus* densities sampled inside the oil slick were 2-fold lower than that outside the slick (Edwards et al., 2011). Cyanobacteria were barely 0.5% of the community and undetected in OSS and CT oil mousses, respectively, from surface water of the nGoM (Liu and Liu, 2013). Our results support these field observations that the exposure to sunlight of oil polluted surface water is more detrimental to these Cyanobacteria.

Dark and light conditions increased the relative abundances of specific bacterial genera. *Thalassobius, Winogradskyella, Alcanivorax, Formosa, Pseudomonas, Eubacterium, Erythrobacter, Natronocella*, and *Coxiella* were generally enhanced in the dark, while *Alteromonas, Marinobacter, Labrenzia, Sandarakinotalea, Bartonella*, and *Halomonas* were clearly associated with sunlight (Figure 5). *Thalassobius* was clearly inhibited by sunlight in all cultures with substrates added, but enhanced during the first 10 days in dark treatments. *Thalassobius* is often associated with bacterial communities in seawater enriched with high-molecular-weight dissolved organic matter or labile peptides (McCarren et al., 2010; Liu et al., 2013). However, it is not reported to degrade petroleum hydrocarbon elsewhere but has been shown to utilize phthalate (Wang et al., 2008; Iwaki et al., 2012). However, it is uncertain as to what components of oil or Corexit it is able to utilize.

*Winogradskyella* was present in the core community of an oil polluted beach in the Gulf of Mexico (Newton et al., 2013) and in sand mesocosms from the beach of the Gulf of Mexico spiked with mixtures of PAHs (Kappell et al., 2014). *Coxiella* was always abundant at the end of the incubation period in dark treatments with oil and/or dispersant. *Coxiella* survive in the environment for long periods of time and are resistant to heat, UV radiation, desiccation, pressure, and oxidative stress (Heinzen and Samuel, 2001). This is somewhat contradictory to our findings because *Coxiella* developed only in the substrate-amended dark treatments (Figure 6B). Possibly, these bacteria may have been stimulated by the degradation products of Corexit and oil under dark conditions. This is the first report of this bacterium related to hydrocarbons in the surface water of the Gulf of Mexico, and the ecology of this organism in the region, particularly its association with oil, dispersant and other environmental pollutants, warrants further study.

*Pseudomonas* and *Alcanivorax* are among the well-studied hydrocarbon degraders and strongly associated with the dark treatments in this study. *Alcanivorax* were the most abundant *Gammaproteobacteria* in the beach sand of Florida (Kostka et al., 2011) during the spill. However, in the oil mousses from the surface water collected at about the same time, *Alcanivorax* and *Pseudomonas* were not abundant (Liu and Liu, 2013; Liu et al., 2013). *Pseudomonas* can utilize a wide range of hydrocarbon substrates (Bacosa et al., 2011, 2013; Bacosa and Inoue, 2015), while *Alcanivorax* are often associated with *n*-alkanes and branched alkane degradation such as pristane and phytane (Hara et al., 2003; Harayama et al., 2004; Head et al., 2006; Yakimov et al., 2007; Kostka et al., 2011; Gutierrez et al., 2013b). In our photooxidation and biodegradation study (Bacosa et al., 2015), these compounds were degraded in the dark but not in the light which might be attributed to *Alcanivorax* and/or *Pseudomonas* (Figure S3B). Alternatively, the strong solar radiation may have inhibited the degraders resulting in the lack of degradation of pristane and phytane. For example, our previous work showed that *Alcanivorax* was not enriched in the oil mousse collected during the oil spill (Liu and Liu, 2013). However, why *Alcanivorax* became abundant at the end of light incubation (Figure 6B), when nearly all hydrocarbons were degraded, remains unclear.

Sunlight enhanced selected phylotypes particularly *Alteromonas, Marinobacter, Labrenzia*, and *Halomonas* (Figure 6). From the sea surface oil slick collected near the DWH during the active phase of oil spill, *Marinobacter* was identified through DNA-stable-isotope probing as a hexadecane degrader, *Alteromonas as* a naphthalene degrader, and *Halomonas* as a phenanthrene degrader (Gutierrez et al., 2013a), so these bacteria may have played a similar role in this study. *Marinobacter* are also capable of utilizing various hydrocarbons as sole carbon and energy sources (Gauthier et al., 1992; Hamdan and Fulmer, 2011; Lamendella et al., 2014). In dark incubation, *Marinobacter* was among the most sensitive to Corexit with nearly 100% reduction in viability and production (Hamdan and Fulmer, 2011). This is consistent with our observation that in dark treatments with added Corexit, *Marinobacter* was barely detected during the first 20 days. Increased *Marinobacter* abundance in light treatment may be possible because of photodegradation of inhibitory Corexit components (Batchu et al., 2014; Glover et al., 2014; Kover et al., 2014).

While some bacteria almost exclusively developed under dark or light during the oil degradation, others thrived in both conditions such as *Roseobacter, Thalassospira, Rhodovulum, Gelria*, and *Deinococcus-Thermus. Roseobacter* is ubiquitous in marine environments and reported to degrade various hydrocarbon substrates such as straight chain alkanes, branched alkanes, cyclic alkanes, and PAHs (Coulon et al., 2007; Lamendella et al., 2014). The activities of these bacteria can be photostimulated by photosynthetically active radiation (PAR) (Ruiz-González et al., 2012). In the northern Gulf of Mexico, members of *Clostridia* were low in surface waters but increased in deep water (King et al., 2013) and were more abundant in salt mash sediment when oil concentrations were low (Beazley et al., 2012). The genus *Gelria*, which has not been reported to be abundant in surface water of the Gulf of Mexico, is known to contain thermophilic, synthrophic, and fermenting species that are often detected in methanogenic consortia under high temperature (Plugge et al., 2002; Jaenicke et al., 2011; Zhou et al., 2012). *Deinoccus-Thermus* along with Clostridia of genus *Gelria* dominated in 27- and 36-days light-dispersant cultures. Mortazavi et al. (2013) reported the presence of *Deinococus-Thermus* (1%) in the intertidal sediments of the Gulf of Mexico. *Deinococcus-Thermus* is composed primarily of bacteria that are resistant to environmental hazards such as ultraviolet and ionizing radiations, oxidizing agents, dessicating conditions, and high temperatures (Henne et al., 2004; Cox and Battista, 2005; Omelchenko et al., 2005; Theodorakopoulos et al., 2013). Thus, strong solar radiation and perhaps high temperature (28–30°C), are main drivers of the dominance of these bacteria in late stages of irradiated dispersant.

Bacteria in surface oil slicks or mousse collected in the field can provide insights into the impact of environmental conditions on bacterial development. To date, only our previous work, Liu and Liu (2013), provides comprehensive information on bacterial community associated with oil mousses (CT and OSS) in the surface water of the nGOM immediately following the spill. The development of bacterial phylotypes in the present incubation is in good agreement with those field samples (Figure S5). For example, high proportions of *Marinobacter, Alteromonas, Erythrobacter, Bartonella, Rhodovulum, Thalassospira*, and *Stappia* were found in these oil mousses. Notably, *Marinobacter* was more abundant in more degraded CT mouse than the less degraded OSS mousse (Liu et al., 2012), while *Alteromonas* was 6-fold more abundant in OSS mousse. This is consistent with our findings that *Alteromonas* predominated over *Marinobacter* during the early stage in irradiated treatments with oil, while *Marinobacter* took over after 20 days when oil was already heavily degraded. *Bartonella* abundance in OSS mousse was 3-fold than that in CT mousse, and it was more than 25% at 20 days in our light-oil incubation. *Rhodovulum* comprised nearly 20% of the community in OSS, and *Thalassospira* was more than 25% in CT mousse. In this study, *Rhodovulum* was remarkably abundant in light-dispersant (40%) while less abundant in dark-oil, and *Thalassospira* was abundant in light-oil (15%). Furthermore, *Erythrobacter* was more abundant in OSS mousse and in the dark incubation.

The bacterial communities in these field oil mousse samples appear to be a combination of bacteria developed in light and dark conditions in the present study, but those in the light were more predominant. This is not surprising as oil mousse is thick slick of floating oil aggregate with unexposed and mostly light exposed portions, while the final concentration of 200 ppm in this study is in the form of an oil sheen entirely exposed to sunlight. The results from this incubation experiment support our previous assertion (Liu and Liu, 2013) that high surface temperature and/or strong sunlight played an important role in the development of bacteria in oil polluted waters in the nGOM. We used surface water with low nutrients (${\text{NO}}_{3}^{-}$, 0.03 μM; ${\text{PO}}_{4}^{-3}$, 0.14 μM) that may have limited bacterial growth and oil or Corexit degradation. The offshore waters of the Gulf of Mexico are oligotrophic in nature, thus, results of this study are most representative of the conditions in oligotrophic open ocean in summer.

In the natural environment, the sea surface is constantly stirred up by wind, oxygenated, and lighter hydrocarbons could have evaporated (Liu et al., 2012; Ryerson et al., 2012; Brakstad et al., 2014). In this study, we sealed the incubation bottles to prevent the loss of volatile components and avoid contamination by bacteria. The remarkable degradation of hydrocarbons within 20 days might have resulted to a decrease in oxygen content at the later part of the experiment, but the large headspace and frequent shaking should ensure an oxygenation regime throughout the incubation. In the current investigation, we tried to optimize the experimental conditions such as the exposure to natural sunlight, and incubation using continuously-flowing seawater under natural temperatures in the northern Gulf of Mexico. Even though we have not totally accounted for other physical and chemical processes affecting the fate of oil, our findings provide preliminary results on the evolution of the microbial communities under the influence of oil, Corexit dispersant and sunlight.

## Conclusions

For the first time, we demonstrated the effect of natural sunlight in the nGoM on microbial communities in the presence of Corexit dispersant, crude oil, and both. Sunlight significantly affected community structure and reduced bacterial diversity in irradiated treatments with Corexit, crude oil, or both. Sunlight selected certain phylotypes such as *Alteromonas, Marinobacter, Labrenzia, Sandarakinotalea, Bartonella*, and *Halomonas*. Dark incubation favored *Thalassobius, Winogradskyella, Alcanivorax, Formosa, Pseudomonas, Eubacterium, Erythrobacter, Natronocella*, and *Coxiella*. Results of this incubation study are consistent with the microbial communities found in oil mousses obtained following the DWH spill. Moreover, this study provides compelling evidence that corroborates our previous findings that strong sunlight is a key driver of microbial community structure (Liu and Liu, 2013). Since we used the surface water assemblages collected from the DWH site in May, and incubated under natural sunlight from May to July, our results are reflective of the conditions during the DWH spill, and more generally of oligotrophic open ocean regions during summer months. This study advances our understanding on how sunlight affects the microbial communities in oil polluted oligotrophic marine surface waters, with implications for their ecological function. Further studies are needed to evaluate the impacts of different concentrations of crude oil and Corexit, weathered oil, and photooxidation metabolites on bacterial communities.

## Author contributions

HB: Conceived, designed and performed the experiment, analyzed data, wrote the paper. ZL: Conceived and designed the experiment, wrote the paper. DE: Conceived and designed the experiment, wrote the paper.

### Conflict of interest statement

The authors declare that the research was conducted in the absence of any commercial or financial relationships that could be construed as a potential conflict of interest.
