# Supplementary material for: Natural Sunlight Shapes Crude Oil-Degrading Bacterial Communities in Northern Gulf of Mexico Surface Waters
Source: Front Microbiol. 2015 Dec 1;6:1325. doi: 10.3389/fmicb.2015.01325 (PMC4664628; doi:10.3389/fmicb.2015.01325)
Supplement: Supplementary file 1 [file Presentation1.PPTX]

## Slide 1
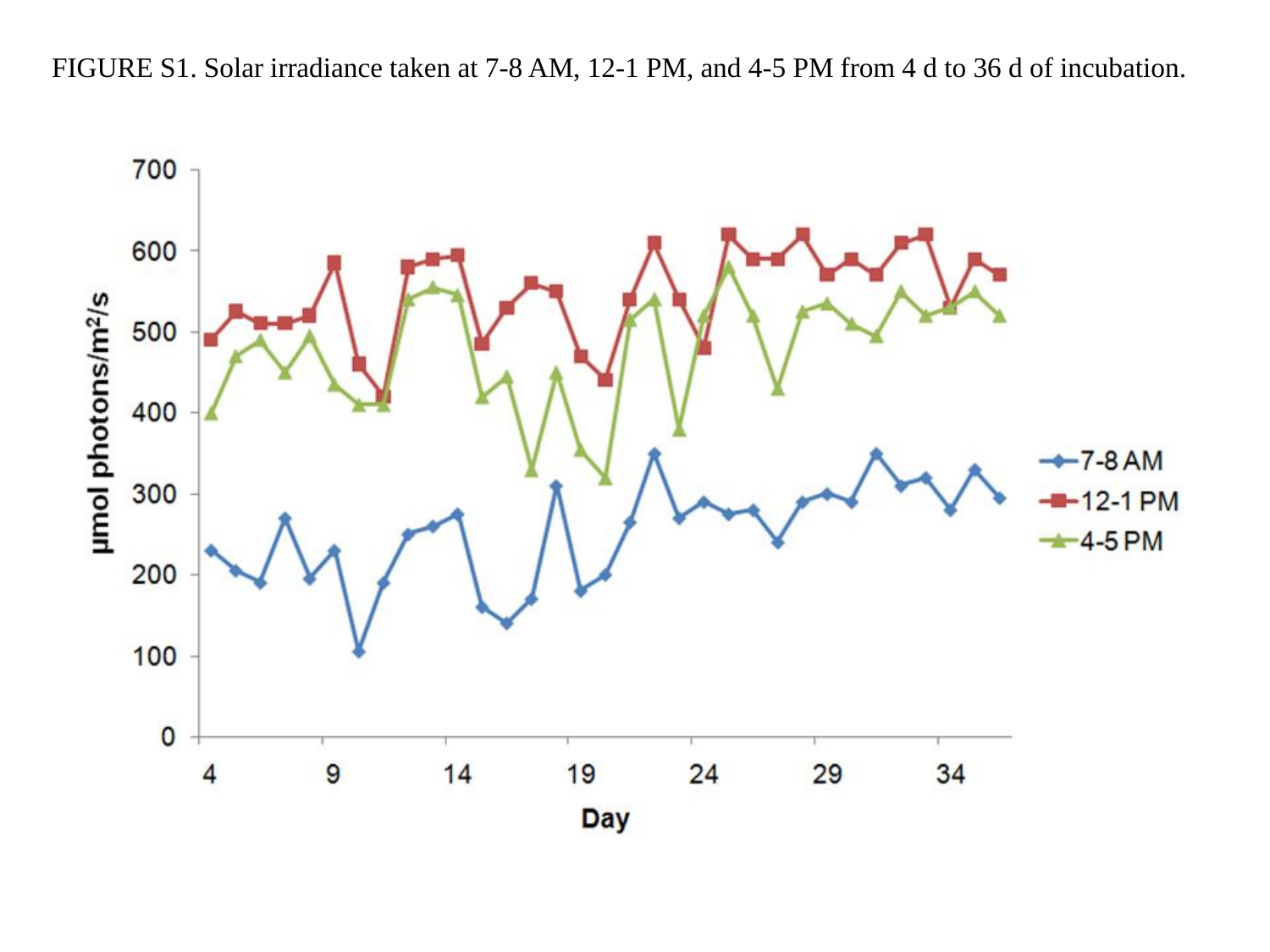

FIGURE S1. Solar irradiance taken at 7-8 AM, 12-1 PM, and 4-5 PM from 4 d to 36 d of incubation.

## Slide 2
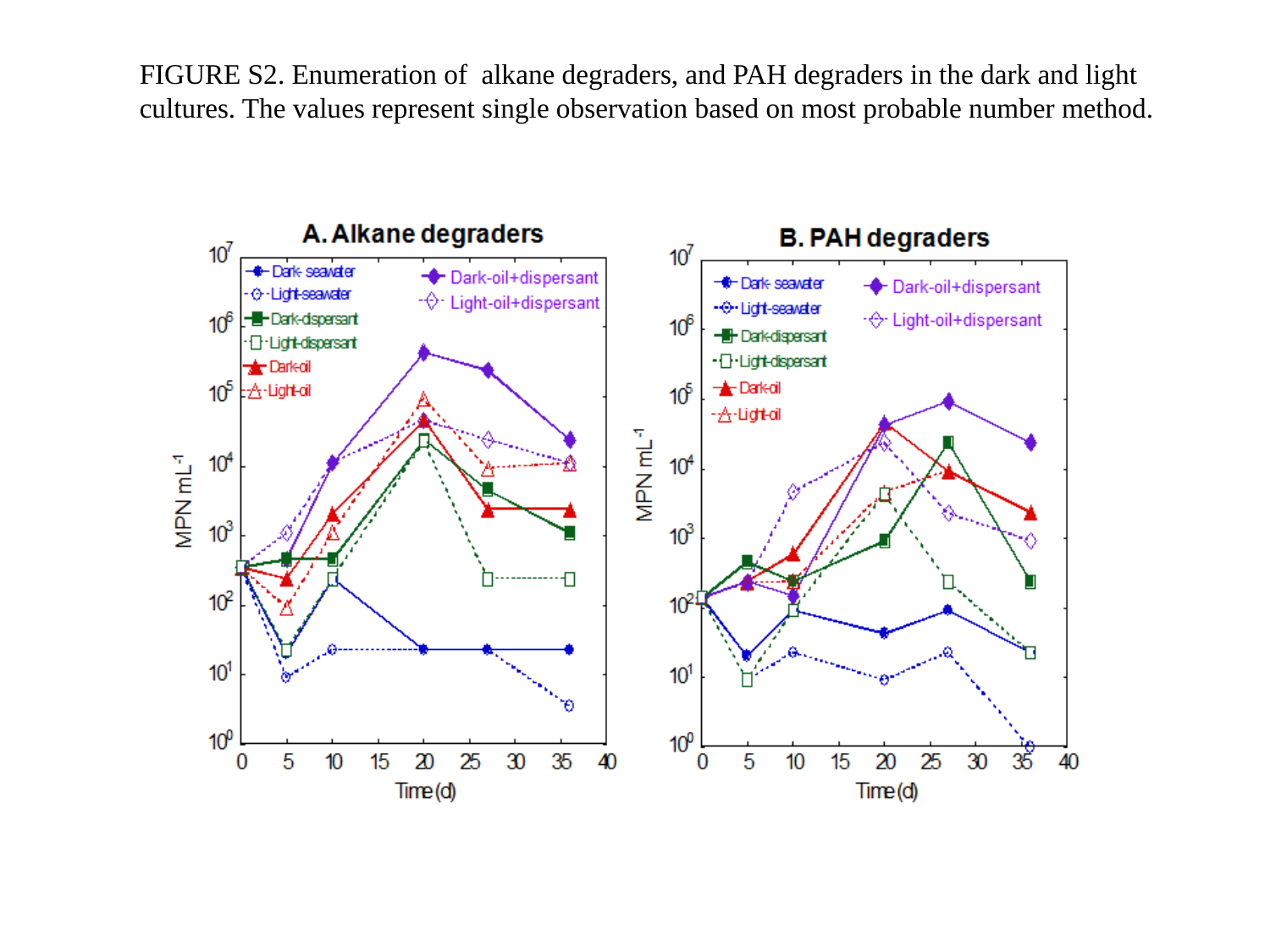

FIGURE S2. Enumeration of alkane degraders, and PAH degraders in the dark and light cultures. The values represent single observation based on most probable number method.

## Slide 3
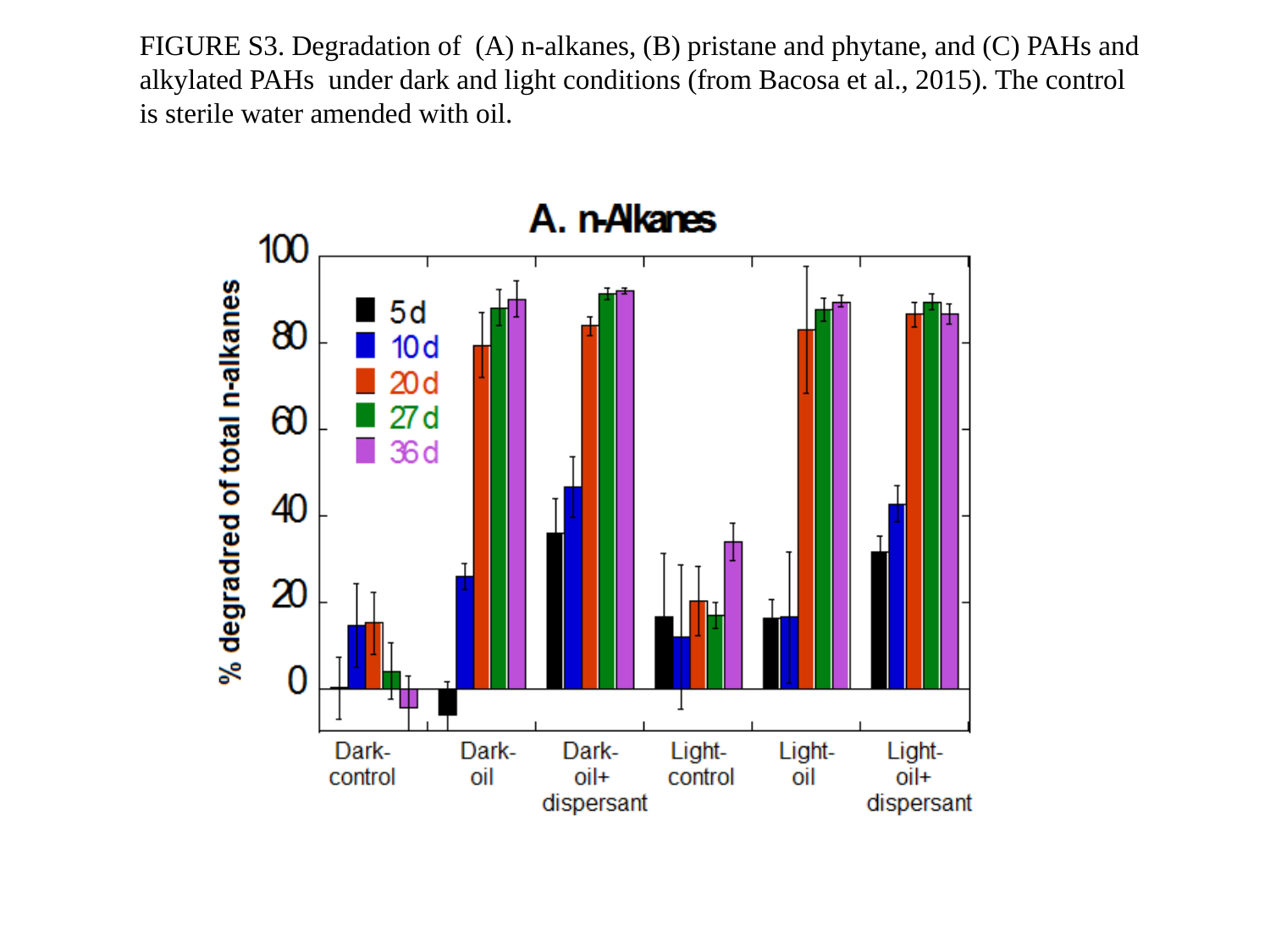

FIGURE S3. Degradation of (A) n-alkanes, (B) pristane and phytane, and (C) PAHs and alkylated PAHs under dark and light conditions (from Bacosa et al., 2015). The control is sterile water amended with oil.

## Slide 4
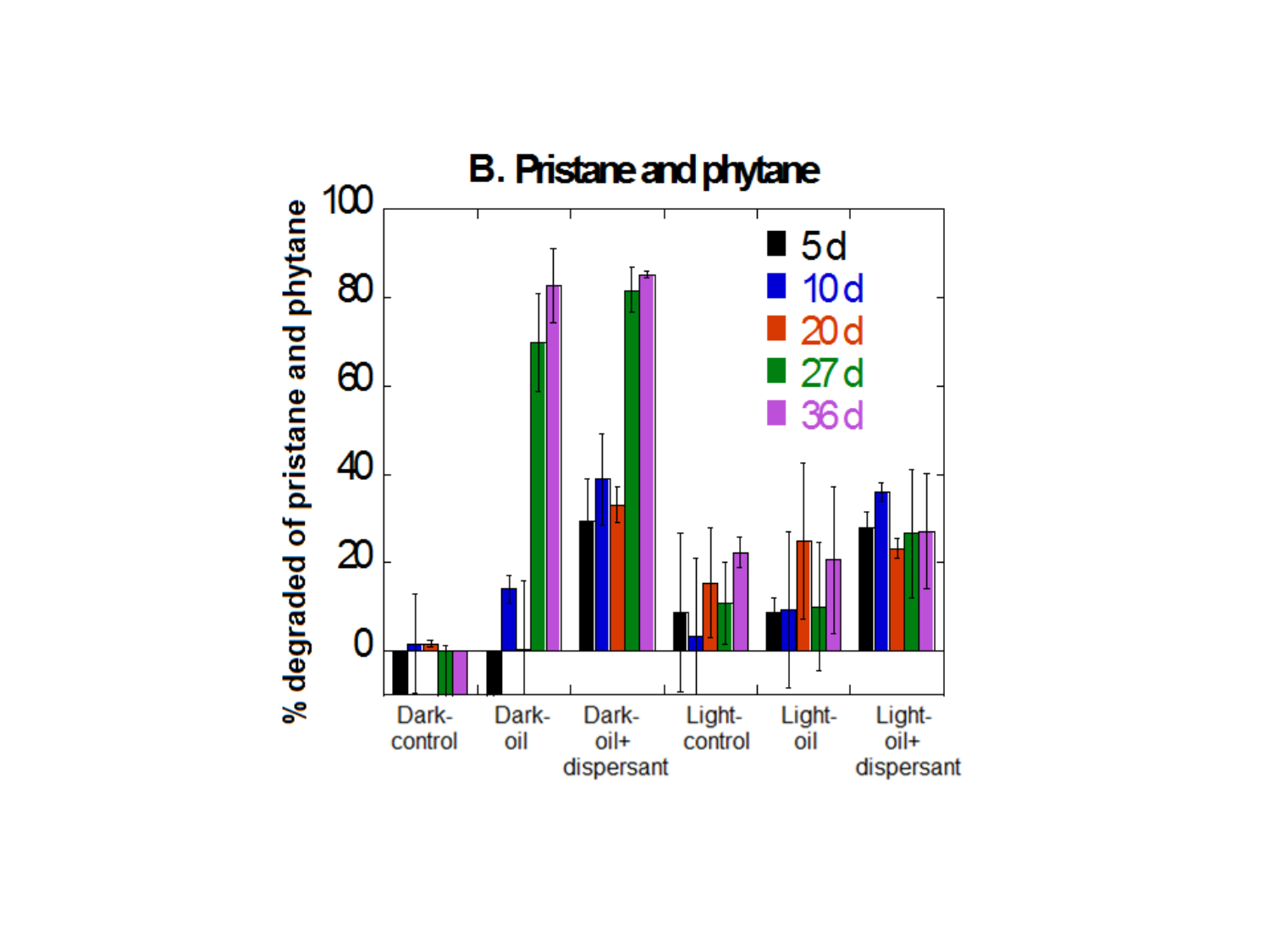

## Slide 5
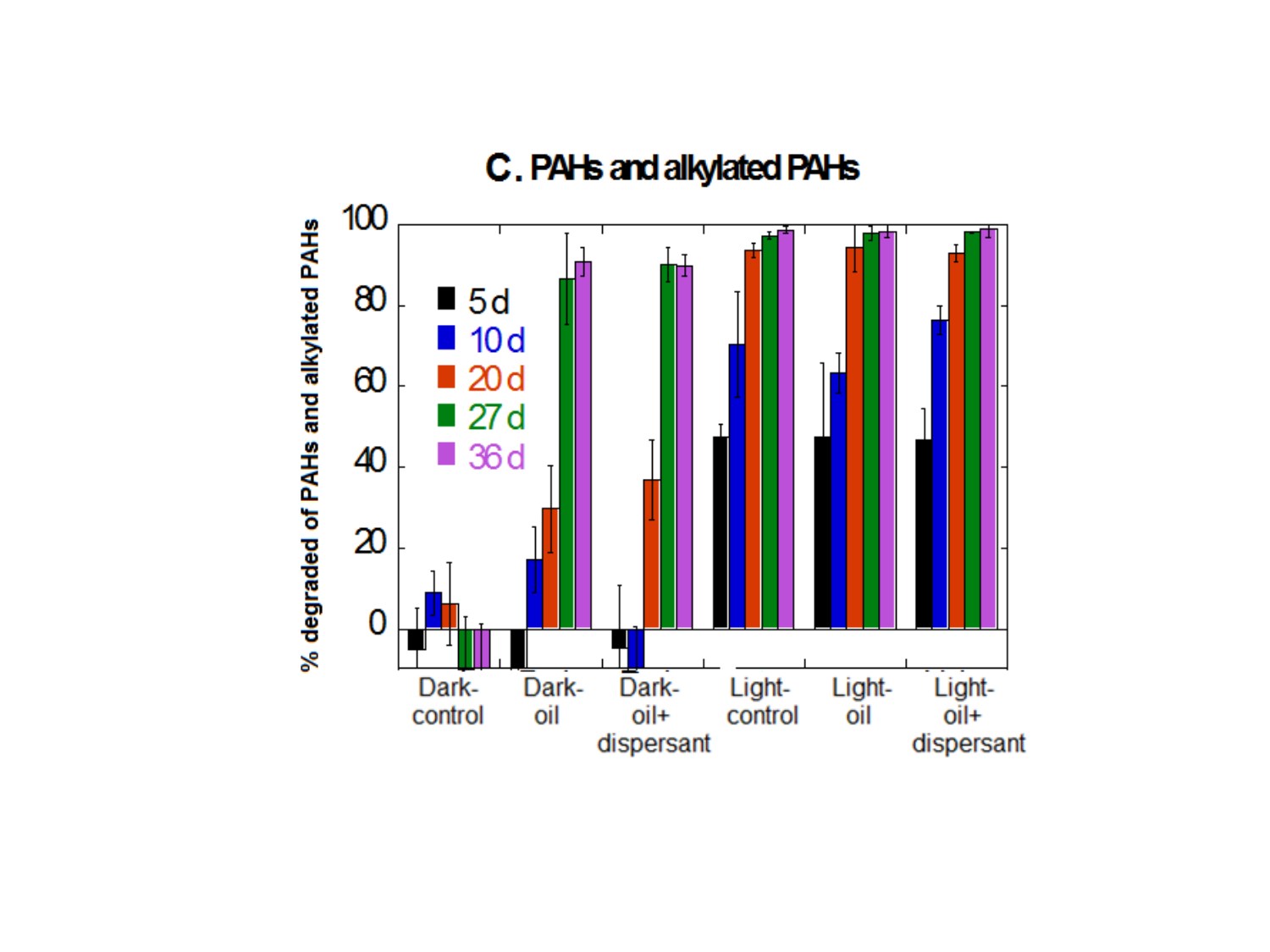

## Slide 6
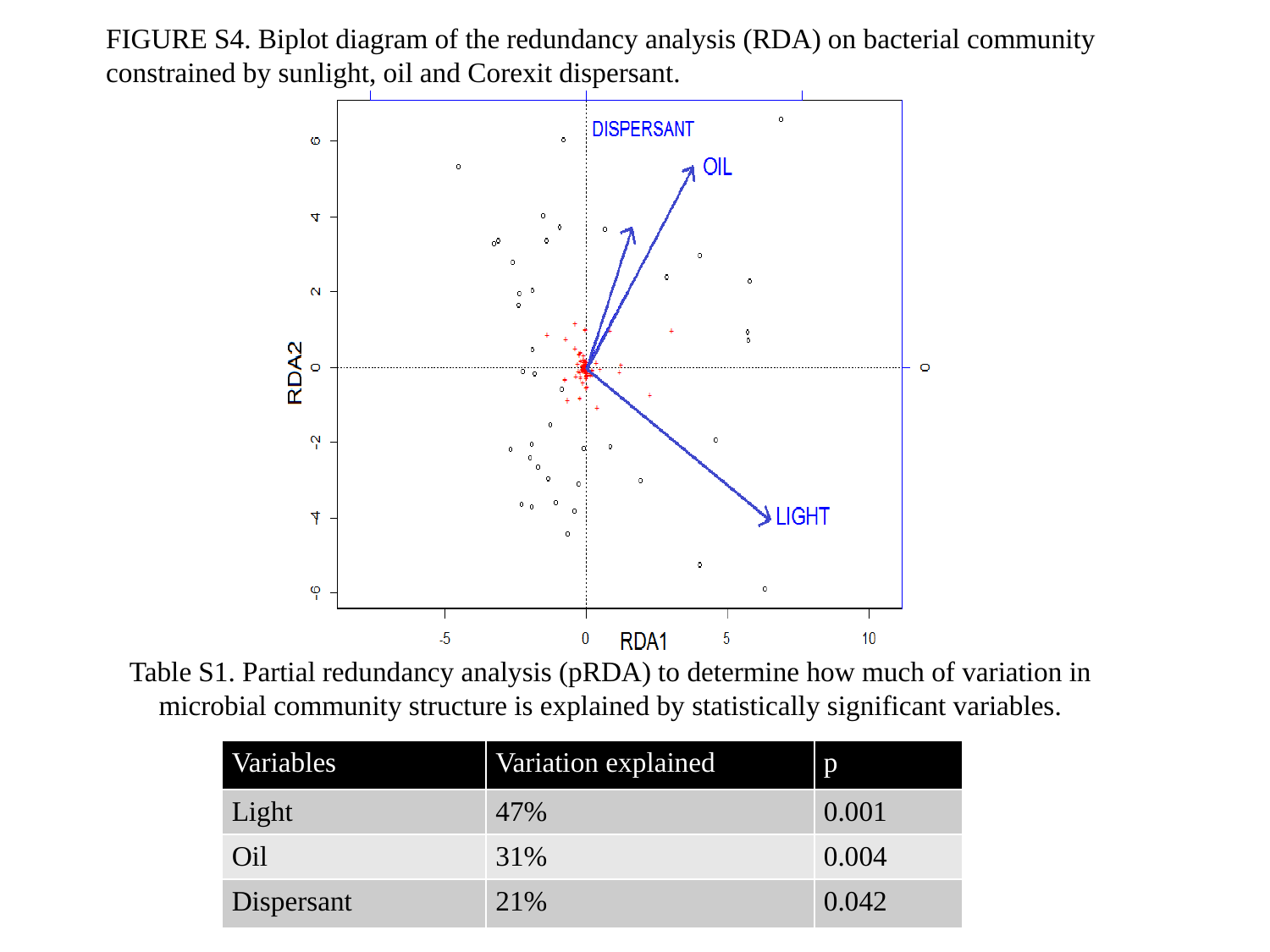

FIGURE S4. Biplot diagram of the redundancy analysis (RDA) on bacterial community constrained by sunlight, oil and Corexit dispersant.
# Table S1. Partial redundancy analysis (pRDA) to determine how much of variation in microbial community structure is explained by statistically significant variables.
| Variables | Variation explained | p |
| --- | --- | --- |
| Light | 47% | 0.001 |
| Oil | 31% | 0.004 |
| Dispersant | 21% | 0.042 |

## Slide 7
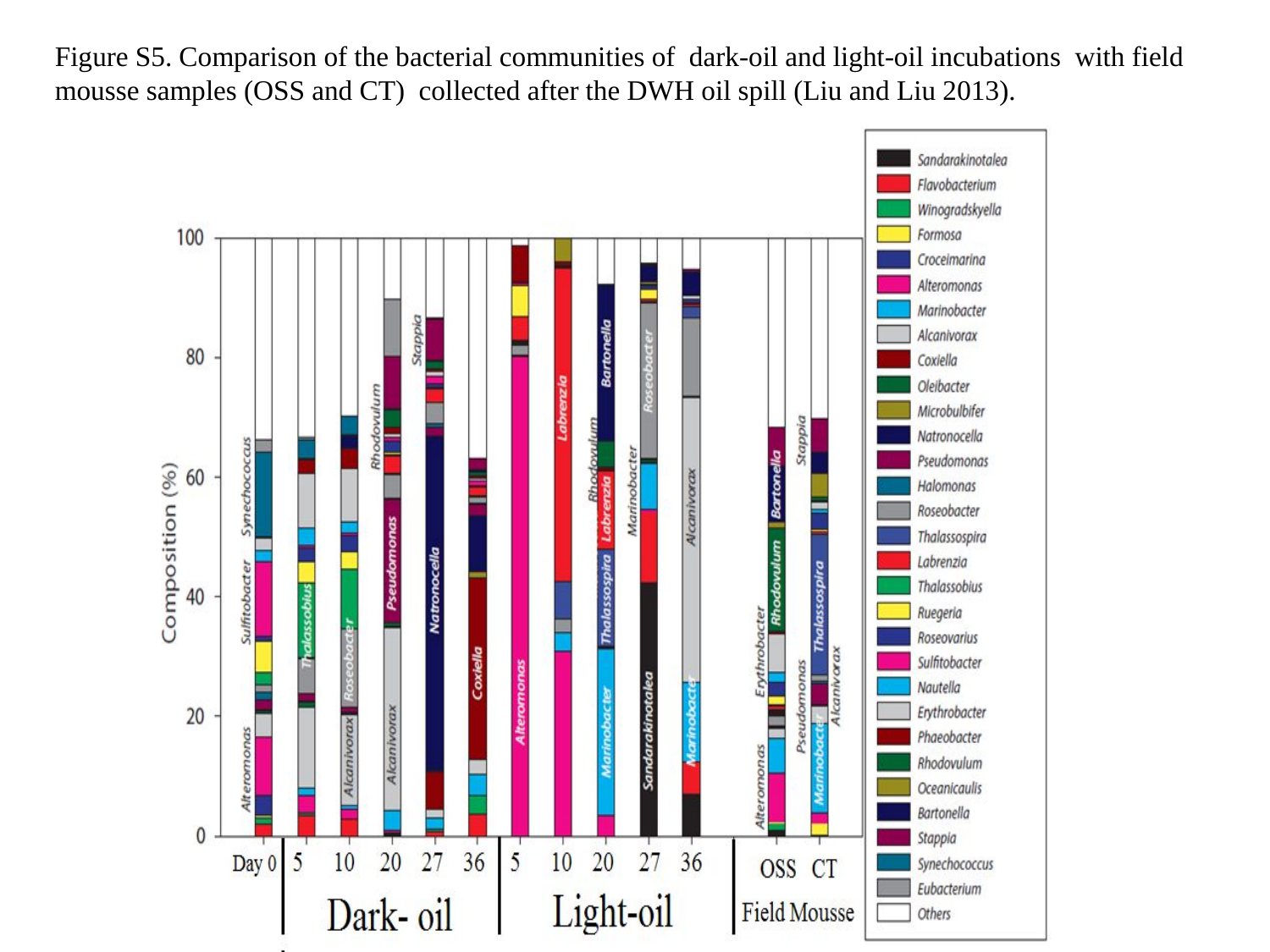

Figure S5. Comparison of the bacterial communities of dark-oil and light-oil incubations with field mousse samples (OSS and CT) collected after the DWH oil spill (Liu and Liu 2013).
